# The diversity of terrestrial isopods in the natural reserve “Saline di Trapani e Paceco” (Crustacea, Isopoda, Oniscidea) in northwestern Sicily

**DOI:** 10.3897/zookeys.176.2367

**Published:** 2012-03-20

**Authors:** Giuseppina Messina, Elisa Pezzino, Giuseppe Montesanto, Domenico Caruso, Bianca Maria Lombardo

**Affiliations:** 1 University of Catania, Department of Biological, Geological and Environmental Sciences, I-95124 Catania, Italy

**Keywords:** Isopoda, Oniscidea, Sicily, biodiversity, frequency, phenology

## Abstract

Ecosystems comprising coastal lakes and ponds are important areas for preserving biodiversity. The natural reserve “Saline di Trapani e Paceco” is an interesting natural area in Sicily, formed by the remaining strips of land among salt pans near the coastline. From January 2008 to January 2010, pitfall trapping was conducted in five sampling sites inside the study area. The community of terrestrial isopods was assessed using the main diversity indices. Twenty-four species were collected, only one of them endemic to western Sicily: *Porcellio siculoccidentalis* Viglianisi, Lombardo & Caruso, 1992. Two species are new to Sicily: *Armadilloniscus candidus* Budde-Lund, 1885 and *Armadilloniscus ellipticus* (Harger, 1878). This is high species richness for a single reserve in Sicily. The extended sampling period also allowed us to study species phenology. Most of the species exhibited higher activity in spring than in autumn while some species also exhibited lower activity in the summer. The species richness revealed that the study area is in an acceptable conservation status; Shannon and Pielou indices also confirmed a more or less even distribution of individuals belonging to different species.

## Introduction

Protected areas have restrictions on human activities aimed at preserving biotic and abiotic components of the landscape. Among the natural protected areas, coastal wetlands are particularly important ecosystems for preserving biodiversity (Adam 1990, Allen and Pye 1992). The natural reserve “Saline di Trapani e Paceco” is one of the most important coastal wetlands in Sicily and an acknowledged Site of Community Importance (SIC), Special Protection Area (ZPS), and “Important Bird Area”; it is among the protected wetlands according to Ramsar Convention. The remaining strips of land are particularly interesting and often very small, acting as banks to the salt pans. Many animal and plant species are endemic to this area (Massa et al. 2006, Grammatico and Fici 2008). Much research has been carried out on bird fauna, vegetation, and fauna in salt pans, but little research has investigated the fauna of the remaining strips of land in between the salt pans (Troia 2008).

The aim of this research was to study the diversity of Oniscidea in the natural reserve “Saline di Trapani e Paceco”. The specific aims were to study the phenology and frequency of the collected species, and to compare species composition and abundance in sites with similar anthropogenic disturbances to determine if there are differences among them. Oniscidean isopods play an important role in terrestrial ecosystems (Sutton 1980). They are found also in such salty environments that are subject to human pressure due to the traditional activity of the salt pans. The salt in sea water is drawn in solid form from the salt pans and extracted for commercial use. One study has been published on Oniscidea community structure inside protected wetland areas in Sicily (Messina et al. 2011). The present study contributes to our knowledge of this area because “the diversity and abundance of terrestrial arthropods can provide a rich base of information to aid efforts in the conservation of biodiversity and the planning and management of nature reserves” (Kremen et al. 1993, Massa and Ingegnoli 1999). In addition, the isopods at the remaining strips of land in front of the shoreline, if these are well preserved, may play an important role in the local food webs. In fact, the various decomposer arthropods that live in these habitats attract the higher-level vertebrate and invertebrate consumers (Chelazzi et al. 1990).

## Material and methods

### Study area

**Figure 1.**
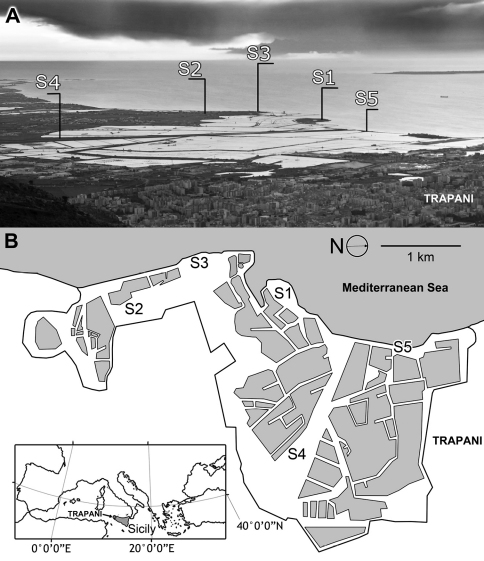
Map of the study area. Sampling sites are indicated. Salt pans are represented by polygons.

The natural reserve “Saline di Trapani e Paceco” (SIC, ITA01007) is located in western Sicily, just south of the town of Trapani (Fig. 1). The reserve has a surface area of 960 ha, consists of a plain characterized by sandy coast with moderate height differences (no more than 5 m above sea level), and is characterized by a large wetland area (80% of the SIC area). The remaining area is divided among areas with intensive human activities (10%), wooded and bushy areas (5%), and agricultural areas (5%). The wetlands are represented by the following categories: groves of reeds, ponds (30 ha), and salt pans (750 ha).

### Sampling

Pitfall trapping was used as the sampling method. This methodology has provided effective sampling also for isopods (Becker 1975, Fleugge and Levens 1977, Al Dabbagh and Block 1981, Caruso and Zetto Brandmayr 1983). The traps were filled with a saturated water/sodium chloride solution. This solution was used to avoid the attractive effects of formalin and vinegar. The salt functions as a preservative. One advantage of pitfall traps is suitable representation of the qualitative and quantitative data for the soil fauna (Sutherland 1996). Pitfall trapping does not favor the capture of all isopods present at a site; therefore, species with low mobility are underestimated. However, this sampling method is the best for invertebrate fauna in the soil (New 1999, Brandmayr et al. 2005). The use of pitfall traps also eliminates the problem of different operator abilities when sampling is done by hand. Sampling was conducted from January 2008 to January 2010 at five sampling sites inside the study area, with a transect of pitfall traps traced in each site. Initially, seven traps were placed in each sampling site. In the data analysis we did not consider the traps always devoid of animals because of disturbances (e.g., grazing, hikers, extreme climatic conditions). The same traps were always empty. Thus, the number of considered traps varied at each sampling site. The distance among the traps was almost 20 meters at each sampling site.

Sampling site S1 (WGS84: 37°59'22.4"N, 012°30'01.0"E) consisted of a small island connected to the land by the embankment of the Morana salt pan. Seven traps were recovered in S1 along a transect orthogonal to the coastline. Sampling site S2 (WGS84: 37°58'13.5"N, 12°30'01.4"E) consisted of strips of land comprising the embankments of the Anselmo salt pan, which consists of an uncultivated area. Six traps were recovered in S2 along a transect parallel to the salt pans. Sampling site S3 (WGS84: 37°58'39.6''N, 12°29'44.7''E) was located along the coast in an uncultivated area along the sea. Five traps were recovered in S3 along a transect parallel to the coastline. Sampling site S4 (WGS84: 37°59'19.6"N, 12°31'27.8"E) consisted of a large uncultivated area bordered by a pond on one side and the Baiata canal on the other side. Six traps were recovered in S4 along a transect orthogonal to a pond that borders the area. Sampling site S5 (WGS84: 38°59'53.7"N, 12°30'29.8"E) consisted of a narrow strip of land that separates the salt pans from the sea, a small island connected to the land by an artificial isthmus, which was made to create the embankments of the salt pans. Five traps were recovered in site S5 along a transect traced between the coastline and salt pans. All of the sites are level and characterized by homogeneous vegetation. Visitors to the reserve, salt pan activity, and grazing are disturbance factors for the sampling sites.

The traps were emptied monthly and the material preserved in 70% ethanol. Sampled individuals were identified in the laboratory and the numbers of males, females, and juveniles were counted.

### Climate data

The study area is characterized by a temperate Mediterranean climate; rain is concentrated during autumn and winter periods, whereas the climate is hot and dry in summer. Data from 1965-1994 indicated an average annual rainfall of 483 mm. The rainiest season is winter (190.1 mm), followed by autumn (176 mm); the rainiest month is December (75.1 mm). The average annual temperature is 18°C, with a maximum temperature of 41.8°C and minimum of 0.1°C.

### Data analysis

The ecological indices used to assess the diversity in each sampling site were: Margalef index (M = S – 1 / lnN, where S is the number of species and N is the total number of individuals), Berger-Parker dominance index (B = N_max_ / N, where N_max_ is the number of individuals of the most abundant species), Shannon- Wiener diversity index (H’), and the Pielou evenness index (J’) (Magurran 1988). Similarities among sites were calculated using Jaccard’s index for presence-absence data and Sörensen’s index for quantitative data. The temporal frequency (F), meaning the ratio between the number of times (months) that the presence of a particular species was observed during the 24 months of sampling, was calculated to describe and summarize data on the presence of certain species during the study period. Thus, this index ranged from 0 (the species has never been observed in a site, but has been encountered in other sites) to 24 (the species was sampled at least once a month for every month of the study). Finally, the temporal frequency was calculated by considering all studied sites, both overlapping and individual. Furthermore, as reported by Fallaci et al. (1994), the species were classified as constant (F ≥ 50%), accessory (25 ≤ F < 50%), accidental (10 ≤ F < 25%), or sporadic (F < 10%). For each species with more than 20 individuals (N), the phenological trend was studied by considering the capture frequency, expressed as a percentage, for each sampling month.

## Results

### Species richness

**Figure 2.**
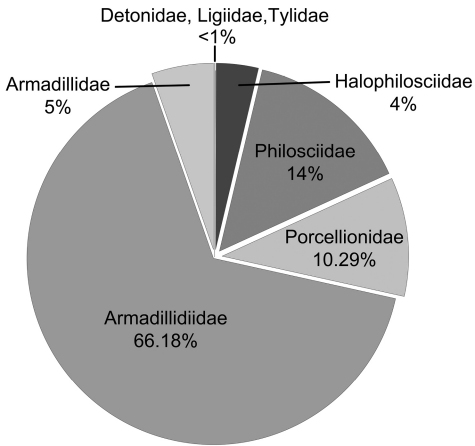
Total frequency (%) of catches for the observed families.

A total of 24,109 isopod specimens were trapped, representing 24 species and 8 families (Table 1). Species of the family Armadillidiidae were the most common (15956, 66.18%), but Philosciidae (3488, 14.47%), Porcellionidae (2482, 10.29%), Armadillidae (1291, 5.35%), and Halophilosciidae (878, 3.64%) were also well represented. The less represented families were Detonidae (6, 0.02%), Tylidae (5, 0.02%), and Ligiidae (3, 0.01%) (Fig. 2). Among these species, only one is endemic, *Porcellio siculoccidentalis* Viglianisi, Lombardo & Caruso, 1992, which can be considered a neo-endemism, found only in western Sicily. Except for *Armadilloniscus candidus* Budde-Lund, 1885 and *Armadilloniscus ellipticus* (Harger, 1878), which were new to the fauna of Sicily, all species collected were already known from the area.

**Table 1.** Species and number of catches for each sampling site; diversity indices values for each sampling site are also reported.

**Species**	**S1**	**S2**	**S3**	**S4**	**S5**	**S1-S5**
**Family Tylidae**						
*Tylos ponticus* Grebnicki, 1874	2				3	5
**Family Ligiidae**						
*Ligia italica* Fabricius, 1798	3					3
**Family Detonidae**						
*Armadilloniscus candidus* Budde-Lund, 1885	1		1		1	3
*Armadilloniscus ellipticus* (Harger, 1878)			2		1	3
**Family Halophilosciidae**						
*Halophiloscia couchii* (Kinahan, 1858)	330	1	520		2	853
*Halophiloscia hirsuta* Verhoeff, 1928			20		1	21
*Stenophiloscia glarearum* Verhoeff, 1908	1		1		2	4
**Family Philosciidae**						
*Chaetophiloscia elongata* (Dollfus, 1884)	218	368	1664	1043	195	3488
**Family Porcellionidae**						
*Porcellionides pruinosus* (Brandt, 1833)				8		8
*Porcellionides sexfasciatus* (Budde-Lund, 1885)		1				1
*Acaeroplastes melanurus* (Budde-Lund, 1885)	28	6	16	12		62
*Agabiformius lentus* (Budde-Lund, 1885)	11	13	21	18	10	73
*Agabiformius obtusus* (Budde-Lund, 1909)				4		4
*Leptotrichus panzerii* (Audouin, 1826)	85	727	357	4	267	1440
*Lucasius pallidus* (Budde-Lund, 1885)	1		1	2		4
*Mica tardus* (Budde-Lund, 1885)	4			4		8
*Porcellio albicornis* (Dollfus,1896)	4	6	10	8	5	33
*Porcellio laevis* Latreille, 1804	66	47	486	178	27	804
*Porcellio siculoccidentalis* Viglianisi, Lombardo, Caruso, 1992	45					45
**Family Armadillidiidae**						
*Armadillidium album* Dollfus, 1887					5	5
*Armadillidium badium* Budde-Lund, 1885	887	23	174	510	2	1596
*Armadillidium decorum* Brandt, 1833	1			424	1	426
*Armadillidium granulatum* Brandt, 1833	14	540	1525	3	11847	13929
**Family Armadillidae**						
*Armadillo officinalis* Dumeril, 1816		7	380	895	9	1291
**Total catch (number of individuals)**	**1701**	**1739**	**5178**	**3113**	**12378**	**24109**
**Species richness**	17	11	15	14	16	24
**Margalef index (M)**	2.1510	1.3400	1.6370	1.6160	1.5920	2.2790
**Shannon-Wiener index (H’)**	1.4980	1.3180	1.7500	1.5750	0.2301	1.4810
**Pielou’s evenness index (J’)**	0.5286	0.5495	0.6460	0.5969	0.0830	0.4661
**Berger-Parker index**	0.5215	0.4181	0.3214	0.3350	0.9571	0.5778

The 24 species found in the present study belong to the following chorological categories. Cosmopolitan: *Porcellionides pruinosus* (Brandt, 1833) and *Porcellio laevis* Latreille, 1804; Mediterranean-Atlantic: *Tylos ponticus* Grebnicki, 1874, *Ligia italica* Fabricius, 1798, *Armadilloniscus ellipticus*, *Halophiloscia couchii* (Kinahan, 1858), *Armadillidium album* Dollfus, 1887, *Armadillidium granulatum* Brandt, 1833, and *Armadillo officinalis* Dumeril, 1816; Holomediterranean: *Chaetophiloscia elongata* (Dollfus, 1884), *Agabiformius lentus* (Budde-Lund, 1885), *Agabiformius obtusus* (Budde-Lund, 1909), and *Leptotrichus panzerii* (Audouin, 1826); North-Mediterranean: *Stenophiloscia glarearum* Verhoeff, 1908 and *Halophiloscia hirsuta* Verhoeff, 1928; West-Mediterranean-Atlantic: *Armadilloniscus candidus*, *Porcellionides sexfasciatus* (Budde-Lund, 1885), *Lucasius pallidus* (Budde-Lund, 1885), and *Acaeroplastes melanurus* (Budde-Lund, 1885); South-Mediterranean: *Mica tardus* (Budde-Lund, 1885) and *Porcellio albicornis* (Dollfus,1896); Calabrian-Sicilian-South-Mediterranean: *Armadillidium badium* Budde-Lund, 1885and *Armadillidium decorum* Brandt, 1833; and endemic species: *Porcellio siculoccidentalis*. (Caruso 1973, Schmalfuss 2003, Taiti and Ferrara 1996).

### Species assemblages

The total number of individuals sampled in each site ranged from a minimum of 1,701 in S1 to a maximum of 12,378 in S5 (Table 1). Species richness at each site ranged from 11 in S2 (M = 1.34) to 17 in S1 (M = 2.15). *Chaetophiloscia elongata* was the dominant species in two sites (S3 and S4) (Table 1). In S3, *Chaetophiloscia elongata* and *Armadillidium granulatum* were co-dominant, whereas a high degree of dominance was restricted to single species in the other sites. *Leptotrichus panzerii* was dominant in S2, *Armadillidium badium* was dominant in S1, and *Armadillidium granulatum* was dominant in S5 (Table 1).

Shannon and Pielou indices had the lowest values in S5 and highest values in S3 (Table 1). Sampling site S3 had few dominant species (H' = 1.75; J' = 0.65) and was represented by 15 species. In S5 (H' = 0.23; J' = 0.08), even though there was greater species richness, high dominance was found due to the massive presence of *Armadillidium granulatum*.

The Jaccard index ranged from 0.43 to 0.72, with sites S3 and S5 being the most similar. The Sörensen index ranged from 0.03 to 0.52. The most similar pair of sites was S1 and S4, and the less similar was S4-S5 (Table 2).

**Table 2.** Similarity analysis based on the Jaccard index (below the diagonal) and Sörensen index (above the diagonal).

	**S1**	**S2**	**S3**	**S4**	**S5**
		0.24	0.27	0.52	0.05
**S2**	0.47		0.40	0.20	0.15
**S3**	0.60	0.63		0.44	0.23
**S4**	0.55	0.56	0.53		0.03
**S5**	0.57	0.50	0.72	0.43	

### Temporal frequency and phenology

Analysis of the temporal frequency in the overall study area showed that 11 species were constant (F ≥ 50%), only one species was accessory (25 ≤ F < 50%), 6 were accidental (10 ≤ F < 25%), and 6 were sporadic (F < 10%). The frequency category for each species did not change in the different sites in regards to the accidental and sporadic species, whereas constant species were not the same among sites (Table 3).

**Table 3.** Temporal frequency (%) analysis for each sampling site.

**S1**		
**Constant**	*Armadillidium badium*	95.65
	*Leptotrichus panzerii*	86.96
	*Chaetophiloscia elongata*	69.57
	*Porcellio laevis*	65.22
	*Porcellio siculoccidentalis*	65.22
	*Halophiloscia couchii*	52.17
**Accessory**	*Acaeroplastes melanurus*	39.13
	*Agabiformius lentus*	26.09
	*Armadillidium granulatum*	26.09
**Accidental**	*Porcellio albicornis*	17.39
**Sporadic**	*Tylos ponticus*	8.70
	*Mica tardus*	8.70
	*Ligia italica*	4.35
	*Armadilloniscus candidus*	4.35
	*Stenophiloscia glarearum*	4.35
	*Lucasius pallidus*	4.35
	*Armadillidium decorum*	4.35
**S2**		
**Constant**	*Leptotrichus panzerii*	91.67
	*Armadillidium granulatum*	79.17
	*Chaetophiloscia elongata*	70.83
	*Porcellio laevis*	54.17
**Accessory**	*Agabiformius lentus*	41.67
	*Armadillidium badium*	37.50
**Accidental**	*Acaeroplastes melanurus*	20.83
	*Armadillo officinalis*	20.83
	*Porcellio albicornis*	16.67
**Sporadic**	*Halophiloscia couchii*	4.17
	*Porcellionides sexfasciatus*	4.17
**S3**		
**Constant**	*Chaetophiloscia elongata*	95.83
	*Porcellio laevis*	95.83
	*Armadillidium granulatum*	95.83
	*Leptotrichus panzerii*	83.33
	*Armadillo officinalis*	70.83
	*Halophiloscia couchii*	66.67
	*Armadillidium badium*	58.33
	*Agabiformius lentus*	54.17
**Accessory**	*Acaeroplastes melanurus*	45.83
**Accidental**	*Porcellio albicornis*	12.50
**Sporadic**	*Halophiloscia hirsuta*	8.33
	*Armadilloniscus candidus*	4.17
	*Armadilloniscus ellipticus*	4.17
	*Stenophiloscia glarearum*	4.17
	*Lucasius pallidus*	4.17
**S4**		
**Constant**	*Chaetophiloscia elongata*	95.83
	*Armadillidium badium*	95.83
	*Armadillidium decorum*	87.50
	*Porcellio laevis*	83.33
	*Armadillo officinalis*	83.33
**Accessory**	*Acaeroplastes melanurus*	37.50
**Accidental**	*Agabiformius lentus*	20.83
**Sporadic**	*Leptotrichus panzerii*	16.67
	*Porcellionides pruinosus*	12.50
	*Porcellio albicornis*	12.50
	*Armadillidium granulatum*	12.50
	*Agabiformius obtusus*	4.17
	*Lucasius pallidus*	4.17
	*Mica tardus*	4.17
**S5**		
**Constant**	*Armadillidium granulatum*	100.00
	*Leptotrichus panzerii*	78.26
	*Porcellio laevis*	56.52
**Accessory**	*Chaetophiloscia elongata*	47.83
	*Armadillo officinalis*	34.78
	*Agabiformius lentus*	26.09
**Accidental**	*Armadillidium album*	17.39
**Sporadic**	*Tylos ponticus*	8.70
	*Halophiloscia couchii*	8.70
	*Porcellio albicornis*	8.70
	*Armadillidium badium*	8.70
	*Armadilloniscus candidus*	4.35
	*Armadilloniscus ellipticus*	4.35
	*Halophiloscia hirsuta*	4.35
	*Stenophiloscia glarearum*	4.35
	*Armadillidium decorum*	4.35
**S1 – S5**		
**Constant**	*Chaetophiloscia elongata*	100.00
	*Porcellio laevis*	100.00
	*Armadillidium granulatum*	100.00
	*Leptotrichus panzerii*	95.83
	*Armadillidium badium*	95.83
	*Armadillidium decorum*	87.50
	*Armadillo officinalis*	87.50
	*Agabiformius lentus*	79.17
	*Halophiloscia couchii*	75.00
	*Acaeroplastes melanurus*	75.00
	*Porcellio siculoccidentalis*	62.50
**Accessory**	*Porcellio albicornis*	41.67
**Accidental**	*Armadillidium album*	16.67
**S1 – S5 continued**		
**Sporadic**	*Tylos ponticus*	12.50
	*Halophiloscia hirsuta*	12.50
	*Stenophiloscia glarearum*	12.50
	*Porcellionides pruinosus*	12.50
	*Mica tardus*	12.50
	*Armadilloniscus ellipticus*	8.33
	*Lucasius pallidus*	8.33
	*Ligia italica*	4.17
	*Armadilloniscus candidus*	4.17
	*Porcellionides sexfasciatus*	4.17
	*Agabiformius obtusus*	4.17

Among the constant species only three exhibited a frequency of 100%: *Chaetophiloscia elongata*, *Porcellio laevis* Latreille, 1804, and *Armadillidium granulatum*. *Porcellio laevis* was constant at all sites (Table 3); *Chaetophiloscia elongata*, *Armadillidium granulatum*, *Leptotrichus panzerii*, *Armadillidium badium*, *Armadillo officinalis*, *Agabiformius lentus* and *Halophiloscia couchii* (Kinahan, 1858) were constant species in S3, though they sometimes changed frequency category in the other sites; *Chaetophiloscia elongata* was accessory in S5, *Armadillidium granulatum* was accessory in S1 and accidental in S4, *Leptotrichus panzerii* was accidental in S4, *Armadillidium badium* was accidental in S2 and sporadic in S5, *Armadillo officinalis* was accidental in S2 and accessory in S5, *Agabiformius lentus* was accidental in S1 and S4 and accessory in S2 and S5, and *Halophiloscia couchii* was sporadic in S2 and S5. Two species, *Porcellio siculoccidentalis* and *Armadillidium decorum* were constant in only one sampling site, S1 and S4, respectively. *Acaeroplastes melanurus* was not constant in any site. The number of sporadic species varied from two in S2 to nine in S5 (Table 3).

**Figure 3.**
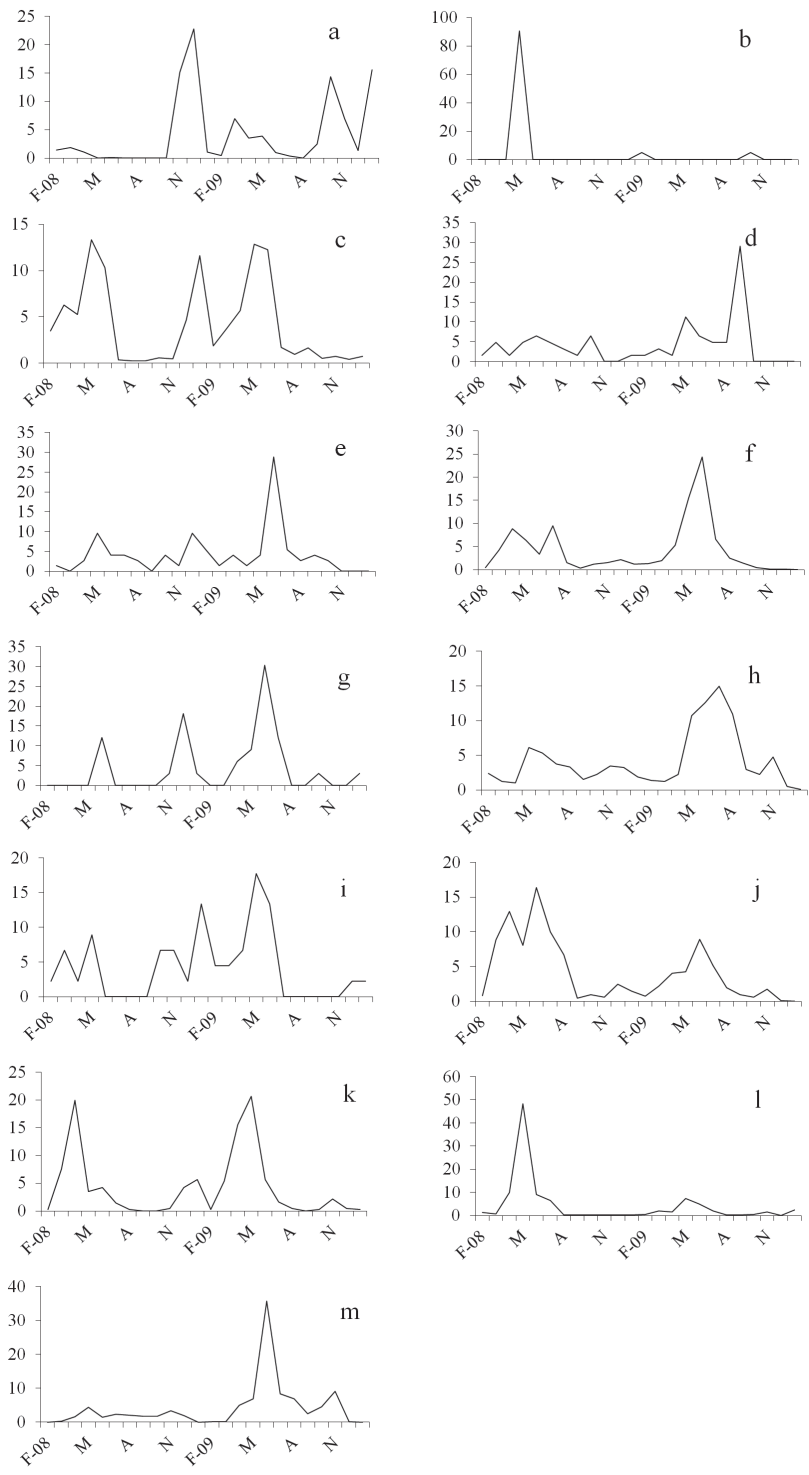
Frequency of catches (%) for each sampling month. **a** *Halophiloscia couchii* (N= 853) **b** *Halophiloscia hirsuta* (N= 21) c *Chaetophiloscia elongata* (N= 3488) **d** *Acaeroplastes melanurus* (N= 62) **e** *Agabiformius lentus* (N= 73) f *Leptotrichus panzerii* (N= 1440) **g** *Porcellio albicornis* (N= 33) **h** *Porcellio laevis* (N= 804) **i** *Porcellio siculoccidentalis* (N= 45) **j** *Armadillidium badium* (N= 1596) **k** *Armadillidium decorum* (N= 426) **l** *Armadillidium granulatum* (N= 13929) **m** *Armadillo officinalis* (N= 1291).

On the basis of the collected species and their ‘abundance’, we were able to evaluate the distribution of species (N >20) in the five sampling sites and to study the activity trend during the sampling period. *Halophiloscia couchii* was represented by a fairly large number of individuals (N=853), which were present more in S1 (38.7%) and S3 (61%) (Table 1). We observed a period of weak activity in the spring with a minimum during the driest periods. During the autumn, the activity intensified with a peak in December that was anticipated in October during the second year (Fig. 3a). *Chaetophiloscia elongata* was represented by a large number of individuals (N=3488), which were found mostly in S3 (48%). The species showed an activity period from February to June in both years. After the summer in which there was no activity, the curve showed a rise with a peak in late autumn (Fig. 3c). *Agabiformius lentus* was represented by a small number of individuals (N=73), which were distributed equally in the five sampling sites. Generally, this species exhibited weak activity that intensified slightly in May and December of the first year and June of the second year (Fig. 3e). *Porcellio albicornis* was represented by only 33 specimens equally distributed in the five sampling sites. The phenological curve showed a peak of activity in late spring, no activity in the summer, and a second smaller peak in autumn (Fig. 3g). *Porcellio siculoccidentalis* was represented by a small number of individuals (N=45) and found only in S1 (Fig. 3i). This species exhibited continuous activity in all months of the year except the driest periods, when it was completely inactive. *Armadillidium badium* was represented by 1596 individuals, many of which were present in S1 (55.6%). The activity of this species started in March, reached a peak in June, and decreased during the summer months. In winter, a second small peak was observed (Fig. 3j). *Armadillidium decorum* was represented by 426 individuals, among which, 424 individuals were present in S4 (99.5%). The phenological curve showed a bimodal trend in both years, with a peak of activity in April and January of the first year and November of the second year (Fig. 3k). *Armadillidium granulatum* was the most represented species with 13,929 individuals, many of which (N=11,847) were collected in S5 (85%). This species showed early activity in March, a peak in late spring, and reduced activity in August (Fig. 3l). *Acaeroplastes melanurus* and *Leptotrichus panzerii* exhibited activity in almost all months of the year, except November and December (Figs. 3d-3f). *Acaeroplastes melanurus* was represented by 62 individuals, many of which were present in S1 (45.2%). *Leptotrichus panzerii* was represented by 1440 individuals, which were collected mostly in S2 (50.5%). *Porcellio laevis* and *Armadillo officinalis* exhibited activity throughout the sampling period with two peaks, in the spring and summer (Figs. 3h-3e). *Porcellio laevis* was represented by 804 individuals, which were collected mostly in S3 (60.4%). *Armadillo officinalis* was represented by 1291 individuals, which were collected mostly in S4 (69.3%) (Fig. 3m).

## Discussion and conclusion

### Species richness

In the study area, we found 27% of the total number of species known in Sicily (90). The 24 species collected were found in very similar habitats, whereas the Sicilian species come from all kinds of biotope (e.g., caves, mountains, woods, etc.). Comparing these data with other research concerning the diversity of terrestrial isopods in the coastal wetland of Vendicari (Natural Reserve in southeastern Sicily, Syracuse province) (Messina et al. 2011), the number of species was nearly the same. Indeed 23 species were found in Vendicari. The two areas have only 13 species in common (*Halophiloscia couchii*, *Halophiloscia hirsuta*, *Chaetophiloscia elongata*, *Porcellionides pruinosus*, *Porcellionides sexfasciatus*, *Acaeroplastes melanurus*, *Agabiformius lentus*, *Agabiformius obtusus*, *Leptotrichus panzerii*, *Porcellio laevis*, *Armadillidium badium*, *Armadillidium granulatum*, and *Armadillo officinalis*). The area of Vendicari seems to be in a better condition than the “Saline di Trapani e Paceco” due to the presence of new species belonging to the genera *Bathytropa*, *Spelaeoniscus*, and *Haplophthalmus*, the former two of which are endemic to the area and belong to genera known to be highly sensitive even to low levels of environmental degradation. Indeed, species belonging to *Bathytropa* and *Spelaeoniscus* live almost exclusively in undisturbed habitats (Caruso and Lombardo 1976).

The species richness in “Saline di Trapani e Paceco” is significantly higher than that of other Mediterranean wetland sites. In coastal wetlands in Tunisia, 14 species were collected (Khemaissia et al. 2011), eight of which (*Ligia italica*, *Chaetophiloscia elongata*, *Porcellionides pruinosus*, *Porcellionides sexfasciatus*, *Leptotrichus panzerii*, *Porcellio laevis*, *Armadillidium granulatum*, and *Armadillo officinalis)* in common with the present study area. In the Berkoukech area (north-west of Tunisia), 12 species of terrestrial isopods were collected (Achouri et al. 2008), five of which (*Chaetophiloscia elongata*, *Porcellionides pruinosus*, *Porcellionides sexfasciatus*, *Leptotrichus panzerii*, and *Armadillidium album*) are also present in our study area. In the Moula-Bouterfess area, 11 species were collected (Hamaïed-Melki et al. 2010), only two of which (*Chaetophiloscia elongata* and *Porcellionides sexfasciatus*) in common with our study. Comparisons with these data, however, are spurious because of different sampling methods.

Considering the ecological requirements of the 24 species, they can be grouped as littoral halophilic (*Tylos ponticus*, *Ligia italica*, *Armadilloniscus candidus*, *Armadilloniscus ellipticus*, *Halophiloscia couchii*, *Halophiloscia hirsuta*, *Stenophiloscia glarearum*, and *Armadillidium album*), coastal (*Acaeroplastes melanurus*, *Agabiformius obtusus*, *Porcellionides sexfasciatus*, and *Armadillidium granulatum*), sabulicolous (*Agabiformius lentus* and *Leptotrichus panzerii*), xerophilic (*Armadillo officinalis*); pratinicolous (*Mica tardus*, *Lucasius pallidus*, *Armadillidium badium*, and *Armadillidium decorum*), humicolous (*Chaetophiloscia elongata* and *Porcellio siculoccidentalis*), anthropophilic (*Porcellionides pruinosus* and *Porcellio laevis*), and myrmecophilous species (*Porcellio albicornis*).

### Species assemblages

All sampling sites except S2 have a high and comparable number of species, but vary in composition. In sampling sites S1, S3, and S5 we found halophilic species whereas in S2 and S4 these species were absent, except for *Halophiloscia couchii*, which was found in S2 at the edge of the salt pans. As indicated by the diversity and evenness indices, a relatively even distribution of individuals among species can be seen in four of the sampling sites. S5 is an exception, due to the very high population of *Armadillidium granulatum*, which is always present with many individuals. Other cases of population explosion are known in the literature (Warburg 1993), such as for *Armadillidium vulgare* (Latreille, 1804)in North America (Hatch 1947) and *Armadillidium granulatum* in Panarea, which covered the streets of the island during the night (Caruso 1968). An enormous population explosion of *Armadillidium decorum* invaded the streets and houses of the town of Collesano (PA) with millions of individuals in the spring of 1998.

Comparing the similarity values (Jaccard index) among the sampling sites, S3 and S5 were qualitatively more similar, having 13 species in common, including strictly halophilic species *Armadilloniscus candidus*, *Armadilloniscus ellipticus*, *Halophiloscia couchii*, *Halophiloscia hirsuta*, and *Stenophiloscia glarearum* and the coastal species *Armadillidium granulatum*. Halophilic species determine the qualitative similarity among all sites. The less similar sites are S4 and S5 (9 species in common) because S4 lacks halophilic species and is richer in species that prefer wet and open areas, such as *Lucasius pallidus* and *Mica tardus*.

The quantitative Sörensen index showed generally low values. S1 and S4 were fairly similar, whereas S4 and S5 were less similar, as for Jaccard’s index.

### Temporal frequency and phenology

Analysis of the temporal frequency of the species in each site showed constancy of species tied to specific habitats. For example, *Armadillidium badium* which lives in grasslands and prefers open areas (Caruso and Lombardo 1982), was constant in S1, where it was found in a large area with low and sparse vegetation, whereas the species *Chaetophiloscia elongata* was constant in S3 and S4, which are both environments with a high level of humidity. A high number of sporadic species was collected in S5; this site is characterized by a narrow strip of land (50 m) between the coastline and salt pans. A majority of the sporadic species are halophilic species typical of the habitats present in this site. Such low frequency values can be explained by the fact that almost all species, including *Armadilloniscus candidus*, *Armadilloniscus ellipticus*, *Halophiloscia couchii*, *Halophiloscia hirsuta*, *Stenophiloscia glarearum*, and *Armadillidium album*, live near the shoreline and rarely move away. The lack of *Tylos ponticus* is strange because migration from the sea to inland and vice versa occurs every night, up to 200 meters from the shoreline (Pardi 1955, Tongiorgi 1969, Alicata et al. 1982).

Most of the species, specifically *Halophiloscia couchii*, *Chaetophiloscia elongata*, *Agabiformius lentus*, *Porcellio albicornis*, *Porcellio siculoccidentalis*, *Armadillidium badium*, *Armadillidium decorum*, and *Armadillidium granulatum*, exhibit high activity in spring and decreased activity during the driest months. A second peak occurs in autumn, perhaps corresponding to the activity of the spring generation. This general trend varies for *Acaeroplastes melanurus*, *Leptotrichus panzerii*, *Porcellio laevis*, and *Armadillo officinalis*, which exhibit low activity in the summer.

The different types of sampling methods used in other studies of similar habitats (Achouri et al. 2008, Hamaïed-Melki et al. 2010, Kemaissia et al. 2011, Hamaïed-Melki et al. 2011), does not permit comparison of phenological data. In agreement with Colombini et al. (2002), though, we found that *Halophiloscia couchii* is more active in April and October. Comparing the results obtained here with those that emerged from similar research carried out in the natural reserve of Vendicari (Messina et al. 2011), we verified that the common species to both areas have an annual activity trend with two peaks in the spring and autumn. The activity periods of *Chaetophiloscia elongata*, *Armadillidium badium*, and *Armadillidium granulatum* do not coincide; in Vendicari *Chaetophiloscia elongata* is most abundant in the summer, whereas *Armadillidium badium* and *Armadillidium granulatum* peak in autumn.

In the present study area, no species of special conservation concern has been found. Nevertheless, and despite the fact that the area is disturbed by human activity at the salt pans, it can be considered of a good environmental quality and of some conservation interest. This conclusion can be inferred by the relatively high number of isopod species and the generally even distribution among them. The only exceptional case was *Armadillidium granulatum* in S5, with a population explosion that could be due among other factors to a drastic decrease in predators due to human activities.
